# Evaluation of the ePlex Blood Culture Identification Panels for Detection of Pathogens in Bloodstream Infections

**DOI:** 10.1128/JCM.01597-18

**Published:** 2019-01-30

**Authors:** Te-Din Huang, Ekaterina Melnik, Pierre Bogaerts, Stephanie Evrard, Youri Glupczynski

**Affiliations:** a Laboratory of Microbiology, CHU UCL Namur, Yvoir, Belgium; Mayo Clinic

**Keywords:** multiplex, sepsis, syndromic

## Abstract

Rapid identification and susceptibility testing results are of importance for the early appropriate therapy of bloodstream infections. The ePlex (GenMark Diagnostics) blood culture identification (BCID) panels are fully automated PCR-based assays designed to identify Gram-positive and Gram-negative bacteria, fungi, and bacterial resistance genes within 1.5 h from positive blood culture. Consecutive non-duplicate positive blood culture episodes were tested by the ePlex system prospectively. The choice of panel(s) (Gram-positive, Gram-negative, and/or fungal pathogens) was defined by Gram-stained microscopy of blood culture-positive bottles (BacT/Alert; bioMérieux). Results with the ePlex panels were compared to the identification results obtained by standard culture-based workflow. In total, 216 positive blood culture episodes were evaluable, yielding 263 identification results. The sensitivity/positive predictive value for detection by the ePlex panels of targeted cultured isolates were 97% and 99% for the Gram-positive panel and 99% and 96% for the Gram-negative panel, resulting in overall agreement rates of 96% and 94% for the Gram-positive and Gram-negative panel, respectively. All 26 samples with targeted resistance results were correctly detected by the ePlex panels. The ePlex panels provided highly accurate results and proved to be an excellent diagnostic tool for the rapid identification of pathogens causing bloodstream infections. The short time to results may be of added value for optimizing the clinical management of patients with sepsis.

## Abstract (precis)

Rapid identification and susceptibility testing results are of importance for the early appropriate therapy of bloodstream infections. The ePlex (GenMark Diagnostics) blood culture identification (BCID) panels are fully automated PCR-based assays designed to identify Gram-positive and Gram-negative bacteria, fungi, and bacterial resistance genes within 1.5 h from positive blood culture.

## INTRODUCTION

Bacteremia and severe sepsis are important causes of mortality in hospitalized patients, especially in those with increasing comorbidities and immunocompromised status (1). Delays in the administration of effective antimicrobial therapy are associated with unfavorable outcomes, especially among patients developing septic shock (2). Nowadays, early empirical treatment may also be ineffective more often than in the past because of the worldwide rise and broadening of bacterial drug resistance.

The implementation of matrix-assisted laser desorption ionization–time of flight mass spectrometry (MALDI-TOF MS) in routine laboratory workflow has accelerated the identification of isolates, but this approach usually requires subculture, potentially generating delays. Direct identification by MALDI-TOF MS on bacterial pellets from blood culture broth has shown efficacy; however, the manual nature of bacterial isolation and the lack of FDA-cleared products for such in-house or commercial methods may impair their routine use (3). Recently, the U.S. Food and Drug Administration (FDA) cleared multiplex molecular assays that can detect a wide range of microorganisms concurrently with specific resistance genes directly from positive blood cultures (BC+), some of which have been made commercially available (4).

The ePlex platform (GenMark Diagnostics, Carlsbad, CA) relies on electrowetting technology to perform multiplexed nucleic acid extraction, amplification, and digestion, followed by the detection of analyte targets using eSensor technology. The ePlex research-use-only (RUO) blood culture identification (BCID) panels are fully automated PCR-based assays designed to identify 20 Gram-positive bacterial genera or species and 4 antimicrobial resistance genes (BCID-GP panel), 21 Gram-negative bacterial genera or species and 6 antimicrobial resistance genes (BCID-GN panel), and 16 fungal genera or species (BCID-FP panel) from positive blood culture. After this study was completed, the ePlex BCID panels have achieved CE-IVD marking (European conformity for *in vitro* diagnostic medical devices); however, here we evaluated the analytical performance of the ePlex BCID panels in the RUO format compared to the routine testing results for the identification of pathogens that cause sepsis and their associated resistance determinants.

## MATERIALS AND METHODS

### Study design.

The evaluation was performed prospectively on consecutive positive blood cultures (BacT/Alert aerobic SA and anaerobic SN bottles; bioMérieux, Marcy-l'Étoile, France) of adult patients sampled from January to October 2017 at CHU UCL Namur Mont-Godinne (CHUMG), a 370-bed tertiary university hospital comprising four intensive care units (total of 30 beds) and with a 120-bed surgical department covering subspecialties including cardiovascular thoracic surgery (lung transplantation), otorhinolaryngo-oncology, and neurosurgery. The CHUMG is part of a larger multisite institution (940 beds) resulting from the association of three hospitals serving a regional population (490,000 inhabitants) in the southern part of Belgium.

The inclusion criteria for enrollment included nonduplicate positive blood culture episodes (one Gram-stained morphotype per septic episode per patient) with organisms visualized at Gram stain microscopy and presumptive clinically significant episodes based on the CDC definition of bloodstream infections (5) (single positive blood culture containing Gram-positive cocci in clusters or diphtheroid Gram-positive rods were excluded). Clinical data were further reviewed to definitively distinguish infection from contamination and to assess the presumed or proven anatomical site source of septicemia (5). The results obtained by the ePlex BCID panels were not reported to clinicians or used for clinical management of the patients.

### Standard microbiological procedures on positive blood culture.

**FIG 1:**
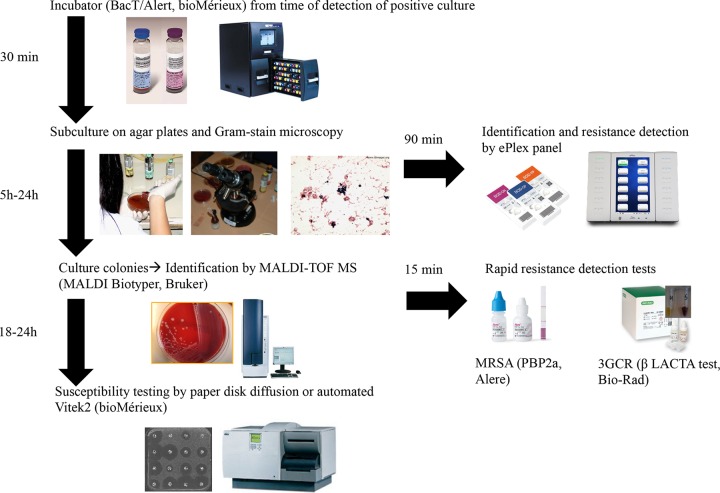
Workflow for identification and susceptibility testing of microorganisms in positive blood cultures.

The routine workflow for inclusion is summarized in Fig. 1. All positive blood culture bottles detected by the BacT/Alert automated blood culture monitoring system immediately underwent subcultures by streaking on nonselective solid agar plates (Trypticase soy agar + 5% sheep blood plate and additional Schaedler agar + 5% sheep blood plate for the anaerobic bottle) from 7 a.m. to midnight. In addition, two aliquots (2 ml each) of the blood culture broth were transferred in sterile microtubes. One tube was used for the ePlex panels (see below), and the other was immediately frozen at −80°C for further analysis and resolution of discrepancies. Gram stain microscopy was performed by dedicated microbiology laboratory technologists, with results electronically transmitted in the laboratory information system and by a phone call during the daytime from 7 a.m. to 7 p.m. Supplementary subcultures of BC+ on selective or differential culture agar plates were performed when different Gram-stained morphotypes were observed by microscopy. Organisms growing from early subcultures (after 5 h of incubation) were identified by matrix-assisted laser desorption ionization time-of-flight mass spectrometry (MALDI-TOF MS) on a MicroFlex LT platform (Bruker Daltonik) using IVD MALDI Biotyper 2.3 software with the database version containing 5,989 entries (6). In addition, the optochin sensitivity test (10 µg optochin; Diatabs; Rosco Diagnostica A/S, Taastrup, Denmark) was used for differentiating Streptococcus pneumoniae from the Streptococcus mitis*/*oralis group.

Rapid detection of clinically important resistance mechanisms was performed on colonies (including those growing on cultures for at least 5 h) by a chromogenic assay (βLACTA test [BLT]; Bio-Rad Laboratories, Marnes-la-Coquette, France) for the detection of resistance to third-generation cephalosporin (3GCR), mostly mediated by extended-spectrum β-lactamase (ESBL) in Enterobacteriaceae species other than natural AmpC producers (7), and by the PBP2a culture colony test (PBP2a; Alere, Scarborough, ME, USA) for the detection of methicillin-resistant Staphylococcus aureus (MRSA) among S. aureus strains (8). Antimicrobial susceptibility testing (AST) was performed by the automated Vitek2 system (bioMérieux, Marcy-l'Étoile, France) for staphylococci (AST-P610 cards), enterococci (AST-P586 cards), and Enterobacteriaceae (AST-N236 cards) and by the disk diffusion method for Gram-negative nonfermenters and for all other bacteria using CLSI guidelines (9). The methicillin resistance phenotype in staphylococci was inferred from a result of resistance to cefoxitin, while vancomycin resistance in enterococci was suspected on the basis of a result of nonsusceptibility to vancomycin. The carriage of ESBL- and/or of carbapenemase-encoding genes in Gram-negative bacterial isolates with suspicious resistance phenotypes (according to CLSI interpretative guidelines) was confirmed by in-house multiplex PCR assays targeting major ESBLs and carbapenemases, followed by amplicon sequencing (10). Vancomycin-resistant enterococci (VRE) were tested for *vanA* and *vanB* genes using the Xpert *vanA*/*vanB* kit on the GeneXpert platform (Cepheid, Sunnyvale, CA, USA) (11).

### ePlex BCID panel testing.

The ePlex BCID panels were performed by a microbiology laboratory technologist during daytime from 7 a.m. to 7 p.m. according to the manufacturers’ instructions. This occurred on the first positive blood culture bottle (SA or SN) from an episode of bacteremia within 1 h after Gram stain microscopy result. The choice of ePlex panel (BCID-GP, BCID-GN, and/or BCID-FP) was based on the Gram stain results. Briefly, 50 µl of the positive bottle broth was dispensed into the cartridge, which was loaded into the ePlex instrument, with results available after approximately 90 min of run time. The pathogens (species and genera) covered for identification and the resistance genes targeted by the three ePlex panels are listed in Table S1 in the supplemental material.

### Data analysis and resolution of discrepancies.

The results from the ePlex system were compared to those obtained by standard procedures. For the identification of the pathogens, an ePlex result was categorized as true positive (TP) if confirmed on growing isolates, while a discrepancy was defined as growing organisms targeted on the ePlex panels which were not detected (false negative [FN]) or as a positive ePlex identification without the corresponding pathogen being detected or identified on culture (false positive [FP]). For the detection of the resistance determinants, a discrepancy was defined as the presence of the targeted resistance mechanism (PBP2a positivity for staphylococci and resistance-encoding genes detected for *Enterococcus* and Gram negatives) on the isolate grown from solid culture but that did not generate a corresponding positive result by the ePlex system (FN) or as the detection of a resistance gene by the ePlex system that could not be detected on any of the growing colonies on agar plates (FP). The discrepancies were further investigated by performing subcultures on frozen discrepant blood culture broth aliquots for isolate recovery and by reviewing the conventional microbiological results of other blood culture samples collected during the same episode of sepsis. An FN ePlex result was eventually changed to true negative (TN) if the isolate identified in the first subculture was not recovered by the secondary subculture. An FP ePlex result was eventually changed to TP if the positive detection was subsequently recovered at secondary subculture or if it was found in at least one other blood culture bottle of the same blood culture episode. The sensitivity of each positive target by the ePlex system was calculated as TP/(TP + FN) and the specificity as TN/(TN + FP), TN being the number of samples tested negative for the target both by the ePlex panel and by culture. The overall sample agreement (both for identification and for detection of resistance determinants) was determined after resolution of discrepancies.

## RESULTS

**FIG 2:**
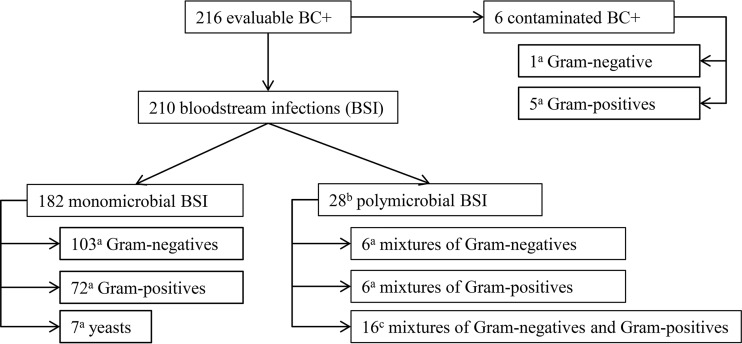
Study flowchart of positive blood culture episodes tested by the ePlex assays. Superscript letters: a, number of corresponding ePlex panels (BCID-GP for GP, BCID-GN for GN, and BCID-FP for yeasts) tested; b, included 20 episodes with multiples species detected within the same bottle tested by ePlex and 8 with different species detected in separate bottles of the same patient; c, included 4 episodes tested with BCID-GP, 5 with BCID-GN, and 7 with both panels.

The distribution of positive blood culture episodes tested by the ePlex assays is presented in a flowchart in Fig. 2. Overall, 210 clinically significant episodes of bloodstream infections (BSI), including 182 monomicrobial and 28 polymicrobial infections, were included in the analysis. Of note, 7 of the BSI were tested by both the BCID-GP and the BCID-GN panels based on microscopy results.

The identification results of organisms targeted by the ePlex BCID panels are shown in Table 1, and the results and resolution of discrepant samples between ePlex and primary subculture are detailed in Table 2. Ninety-seven Gram-positive and 5 Gram-negative (by pan-Gram-negative [Pan-GN] detection) isolates were correctly identified by the BCID-GP panels, while one Listeria monocytogenes detection and one *Lactobacillus* detection were not confirmed by primary subculture of the samples. The BCID-GN panel correctly identified 119 Gram-negative and 7 Gram-positive (by pan-Gram-positive [Pan-GP] detections) isolates, while 4 Fusobacterium necrophorum, 1 Stenotrophomonas maltophilia, 1 Bacteroides fragilis, and 2 Pan-GP detections were not confirmed by primary subculture of the samples. The subsequent subcultures from frozen aliquots that yielded discrepant results did allow recovery of L. monocytogenes, S. maltophilia, and B. fragilis isolates that went unnoticed by initial culture. Results obtained by the ePlex thus were considered true positives for these three isolates. The other 7 positive detections by the ePlex panels not confirmed by secondary subcultures or cultured from the other BC+ bottles of the same patients were called false positives. On the other hand, 3 isolates also recovered in polymicrobial cultures with other organisms were missed by the ePlex (false negatives). One S. epidermidis isolate missed by the BCID-GP panel was considered a contaminant in the setting of an S. aureus BSI. One Escherichia coli isolate was missed by the Pan-GN assay of the BCID-GP panel detecting an Enterococcus faecalis isolate. One Pseudomonas aeruginosa isolate was missed by the BCID-GN in a mixed BSI with E. coli. Regarding fungal organisms, all 6 *Candida* sp. isolates grown by culture and targeted by the ePlex were correctly identified by the BCID-FP panel. One isolate of Candida inconspicua not targeted by the BCID-FP panel was found in one episode of sepsis.

**TABLE 1:** Identification results and performance of detection for organisms targeted by the ePlex BCID panels compared to culture

ePlex panel (no. of cartridges tested with valid results) and positive targets by ePlex	Isolate identification on culture (*n*)	Isolates not detected by ePlex (*n*)	Sensitivity (%)	Specificity (%)
BCID-GP (93)				
*Staphylococcus,* S. aureus	S. aureus (14)		14/14 (100)	79/79 (100)
*Staphylococcus,* S. epidermidis	S. epidermidis (20)	S. epidermidis (1)	20/21 (95)	72/72 (100)
*Staphylococcus*^*b*^	S. haemolyticus (5)		40/40^*b*^ (100)	53/53^*b*^ (100)
*Streptococcus,* S. pyogenes	S. pyogenes (1)		1/1 (100)	92/92 (100)
*Streptococcus,* S. agalactiae	S. agalactiae (3)		3/3 (100)	90/90 (100)
*Streptococcus,* S. pneumoniae	S. pneumoniae (9)		9/9 (100)	84/84 (100)
*Streptococcus*, S. anginosus group	S. anginosus (2), S. intermedius (1)		3/3 (100)	90/90 (100)
*Streptococcus*^*b*^	S. gallolyticus (4), S. dysgalactiae (3), S. mitis (3), S. lutetiensis (1)		27/27^*b*^ (100)	66/66^*b*^ (100)
*Enterococcus,* E. faecalis	E. faecalis (17)		17/17 (100)	76/76 (100)
*Enterococcus,* E. faecium	E. faecium (13)		13/13 (100)	80/80 (100)
*Enterococcus*^*b*^	None		30/30^*b*^ (100)	63/63^*b*^ (100)
*Corynebacterium*	C. mucifaciens (1)		1/1 (100)	92/92 (100)
*Listeria,* L. monocytogenes	L. monocytogenes^*a*^ (1)		1/1 (100)	92/92 (100)
*Lactobacillus*	Not cultured (1)		NA^*c*^	92/93 (99)
Pan-Gram negative	E. coli (3), P. mirabilis (1), Bacteroides caccae (1)	E. coli (1)	5/6 (83)	87/87 (100)
BCID-GN (122)				
E. coli	E. coli (59)		59/59 (100)	63/63 (100)
K. pneumoniae	K. pneumoniae (14)		14/14 (100)	108/108 (100)
K. oxytoca	K. oxytoca (5)		5/5 (100)	117/117 (100)
E. cloacae complex	E. cloacae (5), E. asburiae (1)		6/6 (100)	116/116 (100)
*Enterobacter* (non-*cloacae* complex)	E. aerogenes (3)		3/3 (100)	119/119 (100)
*Citrobacter*	C. koseri (1)		1/1 (100)	121/121 (100)
*Proteus,* P. mirabilis	P. mirabilis (8)		8/8 (100)	114/114 (100)
M. morganii	M. morganii (2)		2/2 (100)	120/120 (100)
*Serratia,* S. marcescens	S. marcescens (3)		3/3 (100)	119/119 (100)
*Serratia*^*b*^	S. liquefaciens (1)		4/4 (100)^*b*^	118/118 (100)^*b*^
P. aeruginosa	P. aeruginosa (13)	P. aeruginosa (1)	13/14 (93)	108/108 (100)
S. maltophilia	S. maltophilia^*a*^ (2)		2/2 (100)	120/120 (100)
B. fragilis	B. fragilis^*a*^ (4)		4/4 (100)	118/118 (100)
F. necrophorum	Not cultured (4)		NA	118/122 (97)
Pan-Gram positive	E. faecalis (3), E. faecium (1), E. avium (1), S. anginosus (1), S. gallolyticus (1), not cultured (2)		7/7 (100)	112/114 (98)
BCID-FP (7)				
C. albicans	C. albicans (2)		2/2 (100)	5/5 (100)
C. glabrata	C. glabrata (3)		3/3 (100)	4/4 (100)
C. parapsilosis	C. parapsilosis (1)		1/1 (100)	6/6 (100)

a Including one isolate recovered at secondary subculture of frozen blood culture sample aliquots.

b Performance calculated including all isolates belonging to the targeted genus.

c NA, not applicable.

**TABLE 2:** Discrepancy resolution of sample results between the ePlex BCID panels and primary subculture^*a*^

Sample no.	Bottle type	Panel tested	Gram stain result	ePlex result	Primary culture result	Secondary culture result	Sample final call
6	Anaerobic	BCID-GP	GPCC	S. aureus, *mecA*	MS S. aureus	MS S. aureus MR S. epidermidis	False negative
24	Aerobic	BCID-GP	GPCP, GNR	E. faecalis Pan-GN L. monocytogenes	E. faecalis E. coli Not cultured	E. faecalis E. coli L. monocytogenes	Agreement
25	Anaerobic	BCID-GN	GNR, GPCP	E. coli Pan-GP B. fragilis	E. coli E. faecium Not cultured F. gonidiaformans	E. coli E. faecium B. fragilis F. gonidiaformans	Agreement
74	Aerobic	BCID-GP	GPCC	S. aureus *Lactobacillus*	MS S. aureus Not cultured	MS S. aureus Not cultured	False positive
87	Anaerobic	BCID-GN	GNR	P. mirabilis Pan-GP	P. mirabilis Not cultured	P. mirabilis Not cultured	False positive
100	Aerobic	BCID-GN	GNR	E. coli Pan-GP	E. coli Not cultured	E. coli Not cultured	False positive
139	Aerobic	BCID-GN	GNR	E. coli	E. coli P. aeruginosa	E. coli P. aeruginosa	False negative
157	Aerobic	BCID-GP	GPCP	E. faecalis	E. faecalis E. coli	E. faecalis E. coli	False negative
163	Aerobic	BCID-GN	GNR	E. coli K. pneumoniae F. necrophorum	E. coli K. pneumoniae Not cultured	E. coli K. pneumoniae Not cultured	False positive
169	Anaerobic	BCID-GN	GNR	K. pneumoniae F. necrophorum	K. pneumoniae Not cultured	K. pneumoniae Not cultured	False positive
173	Aerobic	BCID-GN	GNR	K. pneumoniae F. necrophorum	K. pneumoniae Not cultured	K. pneumoniae Not cultured	False positive
208	Anaerobic	BCID-GN	GNR	F. necrophorum	Not cultured C. ramosum B. caccae	Not cultured C. ramosum B. caccae	False positive
216	Aerobic	BCID-GN	GNR	E. cloacae complex P. aeruginosa S. maltophilia	E. cloacae P. aeruginosa Not cultured A. pittii	E. cloacae P. aeruginosa S. maltophilia A. pittii	Agreement

a Samples containing exclusively isolates not targeted by ePlex were excluded. GPCC, Gram-positive cocci in clusters; GPCP, Gram-positive cocci in pairs/chains; GNR, Gram-negative rods; MR, methicillin resistant; MS, methicillin susceptible.

Sixteen bacterial or fungal pathogens (6%) out of the 256 isolates growing from blood culture episodes were not targeted by the ePlex BCID panels. These organisms are listed in Table 3. Ten out of 16 of the isolates not targeted by the ePlex panels belong to the group of strict anaerobes.

**TABLE 3:** Cultured organisms not targeted by the ePlex BCID panels (*n* = 16; one isolate each per species)

ePlex panel (*n*)	Isolate identification (by MALDI-TOF MS)
BCID-GP (5)	Clostridium ramosum
	Eggerthella lenta
	*Eubacterium* spp.
	Gemella haemolysans
	Lactobacillus delbrueckii
BCID-GN (10)	Acinetobacter lwoffii
	Acinetobacter pittii
	Aeromonas veronii
	Bacteroides thetaiotaomicron
	Bacteroides caccae
	Butyricimonas virosa
	Fusobacterium gonidiaformans
	Leptotrichia trevisanii
	Raoultella ornithinolytica Clostridium ramosum (by Pan-GP)
BCID-FP (1)	Candida inconspicua

The results of resistance genes detected by the ePlex BCID panels are presented in Table 4. Of the 93 BCID-GP valid results, 20 samples yielded a unique *mecA* signal, which was concordant with the primary subcultures in 19 samples growing a methicillin-resistant (MR) *Staphylococcus* isolate. Three MRSA isolates, confirmed by a positive PBP2a test result from colonies, were correctly detected as *mecA*-positive S. aureus by the ePlex, while the other 16 *mecA*-positive samples grew MR coagulase-negative staphylococci associated or not associated with methicillin-susceptible (MS) S. aureus. In one sample which detected S. aureus and *mecA* targets by the ePlex system, only MS S. aureus colonies were detected by primary routine culture. Subsequent subculture of this sample from a frozen aliquot yielded an MR S. epidermidis isolate in small proportion in addition to the predominant MS S. aureus colonies. One polymicrobial sample simultaneously containing MR S. aureus (MRSA) and a vancomycin-resistant E. faecium (VRE) isolate was correctly detected by the ePlex BCID-GP, giving *mecA* and *vanA* signals in addition to the detection of the two species. None of the 72 samples negative for Gram-positive resistance markers by the BCID-GP panel grew any methicillin-resistant *Staphylococcus* or vancomycin-resistant *Enterococcus* isolates. From the 122 BCID-GN valid results, four CTX-M type ESBL-producing E. coli and one Klebsiella oxytoca isolate (all positive by BLT) were also accurately detected by the ePlex system. Of the 117 samples negative for Gram-negative resistance markers by the BCID-GN panel, neither an acquired carbapenem resistance phenotype nor an acquired carbapenemase producer was found, while there was only one 3GCR E. coli isolate which carried an ESBL not targeted by the BCID-GN panel (TEM-52).

**TABLE 4:** Detection results of resistance genes targeted by the ePlex BCID panels (*n* = 26)

Resistance gene(s) detected by ePlex panel	Organism(s) targets detected by ePlex	Resistance phenotype(s) and genotype(s) on cultured isolates^*a*^	*n*
BCID-GP			
*mecA*	*Staphylococcus,* S. epidermidis	MR S. epidermidis	12
	*Staphylococcus*	MR S. haemolyticus	2
	*Staphylococcus,* S. aureus	MR S. aureus	3
	*Staphylococcus,* S. aureus	MS S. aureus (+ MR S. epidermidis^*b*^)	1
	*Staphylococcus,* S. aureus, S. epidermidis	MS S. aureus + MR S. epidermidis + MR S. haemolyticus	1
	*Staphylococcus,* S. epidermidis	MR S. epidermidis + MR S. haemolyticus	1
*mecA* and *vanA*	*Staphylococcus,* S. aureus, E. faecium	MR S. aureus + VR E. faecium (*vanA* positive)	1
BCID-GN			
*bla*_CTX-M_	E. coli	CTX-M group 1 ESBL-producing E. coli	4
	K. oxytoca	CTX-M group 9 ESBL-producing K. oxytoca	1

a MR, methicillin resistant; MS, methicillin susceptible; VR, vancomycin resistant.

b Isolate recovered at secondary subculture of frozen blood culture sample aliquots.

Following exclusion of invalid results and resolution of discrepant results, the performance of detection for each positive target of the ePlex panels compared to culture is detailed in Table 1. Overall sample agreement (panels targeted organism identification and resistance marker detection) between the ePlex assays and standard cultures reached 96% (89/93; 95% confidence interval [CI], 89% to 98%) and 94% (115/122; 95% CI, 89% to 97%) for the BCID-GP and the BCID-GN panels, respectively. The sensitivity for detection by the ePlex system of targeted cultured isolates was 97% (103/105; 95% CI, 93% to 99%) and 99% (128/129; 95% CI, 96% to 100%), while the positive predictive value of organism detection by the ePlex system was 99% (103/104; 95% CI, 95% to 100%) and 96% (128/134; 95% CI, 91% to 98%) for the BCID-GP and the BCID-GN panels, respectively.

## DISCUSSION

Conventional identification and susceptibility testing methods of microorganisms usually require at least 2 days from the time blood cultures turn positive. Assuming that septic patients are usually treated empirically on clinical grounds during the time interval elapsing between collection of blood cultures and time to laboratory results, the additional time needed for availability of culture and susceptibility results further adds to the risk that many patients could be treated inappropriately or unnecessarily with broad-spectrum antimicrobial agents (12). Efforts to shorten the time to availability of results have been made through the implementation of newer, more rapid methods or by the improvement of existing colony-testing methods adapted to be used directly on BC+ samples (13). The rapid identification of pathogens in positive blood cultures of patients with sepsis by MALDI-TOF MS has been extensively investigated using in-house or commercial protein extraction methods or after early growth (following 4 to 5 h of incubation) from subculture on solid medium from BC+ samples (6, 14). The latter method of MALDI-TOF MS identification on young positive subcultures proved to provide reliable identification results on the day of blood culture positivity (6) and has been implemented routinely in our laboratory. Similar adaptations of rapid detection of resistance mechanisms that may have important consequences for the choices of antimicrobial therapy have been proposed for the detection of MRSA and of third-generation cephalosporin-resistant (ESBL-producing) Enterobacteriaceae (7, 8) and were also included in our routine workflow. Several multiplex-based molecular diagnostic assays have been developed to achieve even faster diagnosis of BSI, usually targeting a selected panel of the most relevant pathogens and resistance genes. Among the commercially available systems, FilmArray BCID (BioFire Diagnostics/bioMérieux, Salt Lake City, UT, USA) combines within a single panel targets pathogens belonging to Gram positives, Gram negatives, and yeasts, while Verigene (Nanosphere Inc./Luminex, Northbrook, IL, USA) provides two separate panels for Gram-positive- and Gram-negative-related organisms and resistance gene targets (15). Along the same lines, the ePlex (GenMark Diagnostics, Carlsbad, CA, USA) is a new rapid commercial assay. It is based on a digital microfluidic electrowetting technology combined with the electrochemical detection-based eSensor technology with three separate panels available for Gram positives, Gram negatives, and fungal pathogens, including pan-target coverage on the BCID-GP (pan-Gram-negative and pan-*Candida*) and the BCID-GN (pan-Gram-positive and pan-*Candida*) panels, consisting of targets from opposite panels and some of the most commonly found *Candida* species. These panels have coverage of at least 98%, 95%, and 93%, respectively, of the species encountered in BSI in Belgium (16).

Overall, we found a significant proportion (13%) of polymicrobial infections among the 216 evaluable BC+ episodes. This could be explained in part by the higher proportion of BSI secondary to an intra-abdominal source (26%) in our hospital compared to the national data of BSI in Belgian hospitals (12% of intra-abdominal origin) (16).

In total, we did find invalid results for the BCID-GP and BCID-GN panels, which was related to the RUO format of those panels. However, since the panels were in development at the time of the study and this study’s main objective was to assess the analytical performance of the panels, the validity cannot be accurately assessed. Nevertheless, it should be highlighted that a much lower rate of invalid results was observed with the two most recent manufactured lots than with the first two lots of the BCID-GP and BCID-GN cartridges (data not shown). This positive trend suggests great improvement in the robustness of the assay that would be foreseen in its upcoming CE-IVD format, which has <5% invalid rates according to the manufacturer’s guidelines.

Regarding the identification of the bacterial and fungal pathogens associated with sepsis, complete agreement was observed for 234 isolates out of the 263 identification results (89%), while 16 (6%) pathogens grown by culture were not targeted by the ePlex panels. Such a proportion of untargeted microorganisms was in line with the expected coverage based on the national BSI data (16). The species diversity of the 16 isolates not targeted by the ePlex assay (each belonging to different species) suggested good coverage of the BCID panels without missing particular species that would be most frequently encountered in BSI episodes in our setting.

Among the 13 discrepancies when comparing the ePlex panels to the first BC+ subculture results (Table 2), it should be noted that all were encountered in samples already growing at least one other isolate and that more than half (8/13) were in confirmed polymicrobial BSI (two or more species cultured). Subsequent reprocessing from frozen aliquots allowed the recovery of three isolates that had not been detected on the first subculture, highlighting the potential benefit of the ePlex system in the setting of polymicrobial sepsis. Of note, these 3 isolates as well as the 3 isolates undetected by the ePlex panels (also in polymicrobial BC+ samples) were neither visualized nor differentiated from the other bacteria by Gram stain microscopy and appeared in a smaller proportion than the other more predominant species, which might explain the lack of detection by one of the two methods.

The Pan-GN and Pan-GP assays were able to detect 12 isolates growing from Gram-positive and Gram-negative mixed infections, including 4 Gram positives (by Pan-GP) that were not visualized by microscopic examination. The inclusion of these Pan targets (not present on other commercial broad-range multiplex PCR systems) could be of particular added clinical value, because they overcome the disadvantage of the system linking the choice of the panel to test and the Gram stain results, which requires skilled technologists who might not be available everywhere or at all times (especially during off hours). The positivity of these Pan-GN and Pan-GP targets would trigger testing of the complementary ePlex panel on the same BC+ samples and may lead to adjusted therapy targeting the pathogens (bacteria or fungi) belonging to the other group that would not be covered if the treatment was based solely on microscopy. Other experiences did support the utility of multiplex molecular testing (containing targets of both Gram-stained groups) in better and faster recognition of polymicrobial BSI than microscopy (17).

Finally, positive detection on the ePlex could not be confirmed by primary culture or by secondary subculture of frozen aliquots in seven episodes (F. necrophorum [*n* = 4], Pan-GP [*n* = 2], and *Lactobacillus* [*n* = 1]). Although we did not attempt to perform additional molecular detection methods, these 7 positive detections called by the RUO software were analyzed by the manufacturer, indicating that all but one (F. necrophorum in a polymicrobial anaerobe BSI) would be true-negative results if the samples were assayed with the current version of released CE-IVD software (Adam Thornberg, GenMark Diagnostics, personal communication). Updates to the thresholds of these RUO assays in the interpretative software could have avoided the occurrence of some false positives, but such modifications of thresholds were not available during the time of our evaluation and therefore could not be considered.

Regarding the detection of resistance markers, no discrepancy was observed, as all samples with resistance markers detected by the ePlex system were correctly confirmed in cultured isolates (no false positives), and no isolates carrying targeted resistance genes were missed by the ePlex system (no false negatives). Out of the 12 monomicrobial BC+ samples with S. aureus, 9 were *mecA* negative and could have triggered earlier de-escalation from empirical glycopeptide to treatment with narrower-spectrum isoxazolyl penicillins. The simultaneous detection of MRSA and of *vanA*-positive VRE also could have led to earlier administration of antibiotics targeting these multidrug-resistant organisms (MDRO), such as glycylcyclines or oxazolidinones. For Gram negatives, no carbapenemase-producing organisms causing BSI were found during the study period. The observation was in line with the epidemiological setting in our institution (very low proportion of carbapenemase producers among clinical isolates [<1%]; unpublished personal data) and does not support the systematic use of rapid tests for the detection of carbapenemase in our routine workflow. In Enterobacteriaceae, all 5 CTX-M type ESBLs were detected by the ePlex system, but one ESBL family type (TEM-ESBL) not targeted by the BCID-GN panel was positive by BLT. Since the ePlex BCID-GN panel only targets selected resistance markers (on the basis of their prevalence and/or epidemiological importance) among Gram negatives, phenotypical hydrolysis-based tests for the rapid detection of other resistance enzymes could be of complementary value (18), and standardized antimicrobial susceptibility testing is still warranted to detect other (including non-enzyme-mediated) resistance mechanisms and for the final choice of active drugs. The impact of molecularly based diagnostic systems on adjustment of antimicrobial treatment has been shown in other studies, mostly highlighting its benefit for GP bacteremia (19, 20).

After discrepant resolution, we found excellent performance of the ePlex BCID-GP and BCID-GN panels, with sample agreement, sensitivity, and positive predictive values of targeted species being found in 94% or more episodes for both panels. The vast majority of positive targets (32/35) had individual sensitivity of ≥95%, while all but 3 targets (F. necrophorum, *Lactobacillus,* and Pan-GP) showed perfect specificity. This excellent performance was in line with that achieved by other multiplex detection methods for BSI evaluated, with reported sensitivity/specificity ranging from 80% to 97%/91% to 100% for FilmArray BCID panel and 89% to 100%/93% to 100% for the two Verigene BC panels (15). Compared to these commercial diagnostic systems, the main difference and advantage of the ePlex system would be the larger number of targets. Applied to the microorganisms detected in our study, only two *Acinetobacter* species isolates of the 15 isolates not targeted by the ePlex panels were covered by the Verigene BC-GN panel. Advantageous to this study were the four *Serratia* sp., four B. fragilis, two S. maltophilia, and one *Citrobacter* sp. isolates identified by the ePlex system that would have gone undetected by one or both of the two other commercial systems. Further, the ability (not present in FilmArray BCID) to distinguish the S. anginosus group and E. faecium from E. faecalis is appreciated for the added information on the putative source of the BSI or for highlighting the intrinsic resistance of the identified species. For resistance marker detection, the 5 CTX-M ESBL producers would have been missed by FilmArray BCID, which targets only KPC resistance genes.

The small number (*n* = 7) of fungemia detections in our study prevents us from drawing conclusions about the performance of the BCID-FP panel for the detection of fungal pathogens associated with sepsis. However, one Candida inconspicua isolate identified by MALDI-TOF MS (21), a fluconazole-resistant yeast as an emerging agent of invasive infection (22), was not targeted by the FP panel (23). However, there are other *Candida* species of clinical importance, such as C. auris, an emerging multidrug-resistant pathogenic species causing health care-associated infections and outbreaks (24, 25) that GenMark Dx has added to the CE-IVD ePlex BCID-FP panel.

The major advantage of the use of ePlex panels is its intrinsically shorter time to results following BC positivity (2 h) compared to those obtained on subculture (5 h for the fastest-growing organisms) with expected substantial gain, especially in polymicrobial samples (usually requiring additional subculturing steps to achieve full results). The multiplex character of the ePlex also offers advantages for detecting polymicrobial infections in settings such as postoperative complicated abdominal infections. However, the main limit of our assessment is the observational nature of the study design without modification of our current routine workflow (microbiology workup and ePlex testing during the same daytime work hours). This prevented us from determining precisely the differences in time to results that could have been spared by the use of ePlex panels. We believe the increased speed for results could further be improved with the implementation of the system in a 24-h/7-day workflow, but this would need an appropriate antimicrobial stewardship program applied continuously to achieve significant clinical impact (26).

In conclusion, we found that the ePlex BCID panels proved to achieve excellent accuracy and appeared very convenient for routine use (27) with minimal hands-on time (<2 min). The system is able to provide faster results with potential clinical added value and can serve as a complementary tool to culture, which remains necessary to obtain complete susceptibility results for definitive therapy. Gram stain microscopy to choose the panels to test is required, but the inclusion of Pan targets can help alleviate this drawback. There are few pathogens currently not targeted by the panels, including possible emerging pathogens of clinical importance with increasing resistance to antimicrobials, questioning the versatility of the system for allowing adaptation of some of the panels. Further studies should be carried out in settings with higher prevalence of MDRO, and clinical indicators (outcome, duration of hospitalization, duration of antimicrobial therapy, etc.) and cost-benefit parameters should also be evaluated in different settings.

## Supplementary Material

Supplemental file 1
