# Supplementary material for: Evaluation of the ePlex Blood Culture Identification Panels for Detection of Pathogens in Bloodstream Infections
Source: J Clin Microbiol. 2019 Jan 30;57(2):e01597-18. doi: 10.1128/JCM.01597-18 (PMC6355516; doi:10.1128/JCM.01597-18)
Supplement: Supplemental file 1 [file a1d75fbc83b4d2c90daa9ae2f0b9af98_JCM.01597-18-s0001.pdf]

Table S1. Microorganisms and resistance markers targeted by the ePlex RUO BCID panels

Targets	BCID-GP panel	BCID-GN panel	BCID-FP panel [a]
Organisms	*Bacillus cereus* group<br>*Bacillus subtilis* group<br>*Corynebacterium*<br>*Cutibacterium acnes*<br>*Enterococcus faecalis*<br>*Enterococcus faecium*<br>*Enterococcus*<br>*Lactobacillus (casei/rhamnosus)*<br>*Listeria monocytogenes*<br>*Listeria*<br>*Micrococcus*<br>*Staphylococcus aureus*<br>*Staphylococcus epidermidis*<br>*Staphylococcus lugdunensis*<br>*Staphylococcus*<br>*Streptococcus pyogenes*<br>*Streptococcus agalactiae*<br>*Streptococcus pneumoniae*<br>*Streptococcus anginosus* group<br>*Streptococcus*<br>Pan Gram-negative<br>Pan *Candida*	*Acinetobacter baumannii*<br>*Bacteroides fragilis*<br>*Citrobacter*<br>*Cronobacter sakazakii*<br>*Enterobacter cloacae* complex<br>*Enterobacter* non-cloacae complex<br>*Escherichia coli*<br>*Fusobacterium necrophorum*<br>*Fusobacterium nucleatum*<br>*Haemophilus influenzae*<br>*Klebsiella oxytoca*<br>*Klebsiella pneumoniae*<br>*Morganella morganii*<br>*Neisseria meningitidis*<br>*Proteus mirabilis*<br>*Proteus*<br>*Pseudomonas aeruginosa*<br>*Salmonella*<br>*Serratia marcescens*<br>*Serratia*<br>*Stenotrophomonas maltophilia*<br>Pan Gram-positive<br>Pan *Candida*	*Candida albicans*<br>*Candida dubliniensis*<br>*Candida famata*<br>*Candida glabrata*<br>*Candida guilliermondii*<br>*Candida kefyr*<br>*Candida krusei*<br>*Candida lusitaniae*<br>*Candida parapsilosis*<br>*Candida tropicalis*<br>*Cryptococcus gattii*<br>*Cryptococcus neoformans*<br>*Fusarium*<br>*Malassezia furfur*<br>*Rhodotorula*<br>*Trichosporon*
Resistance markers	*mecA*<br>*mecC*<br>*vanA*<br>*vanB*	$bla_{\text{CTX-M}}$<br>bla_{NDM}<br>bla_{VIM}<br>bla_{IMP}	

		bla$_{KPC}$<br>*bla*$_{OXA\ (OXA\text{-}23\ or\ OXA\text{-}48)}$	

[a] CE-IVD ePlex BCID-FP panel does not include *M. furfur*, *Trichosporon* but does include *Candida auris*
